# Effectiveness and Safety of Targeted Agents Combined With Chemoradiotherapy for the Treatment of Esophageal Cancer: A Network Meta-Analysis

**DOI:** 10.3389/fonc.2021.621917

**Published:** 2021-11-29

**Authors:** Peng Liu, Guo-Fei Wang, Hua Peng, Lei Zhang, Xiao-Yan Li, Qiao-Miao Zeng, Qian Li, Jian-Hui Zhou

**Affiliations:** ^1^ Intensive Care Unit of Cardiovascular Surgery Department, Xiangya Hospital Central South University, Changsha, China; ^2^ Department of Nursing, Xiangya Hospital Central South University, Changsha, China; ^3^ Department of Thoracic Surgery, Xiangya Hospital Central South University, Changsha, China; ^4^ Department of Oncological Radiotherapy, Xiangya Hospital Central South University, Changsha, China; ^5^ National Clinical Research Center for Geriatric Disorders, Xiangya Hospital, Changsha, China

**Keywords:** esophageal cancer, targeted agents, chemoradiotherapy, meta-analysis, systematic review

## Abstract

**Background:**

Concurrent chemoradiotherapy (CRT) is the preferred treatment strategy for inoperable esophageal cancer (EC). However, the effect of CRT needs to be improved.

**Methods:**

This study comprehensively analyzed targeted agents combined with CRT for the treatment of EC by a network meta-analysis. The search was performed in public databases from incipient to 5 August 2021. Randomized controlled trials comparing the effect of targeted agents combined with CRT and CRT alone on EC patients were included.

**Results:**

Ten studies were included. For progression-free survival (PFS), nivolumab (67.4%) and erlotinib (64.6%) had advantages based on Cox analysis. Regarding the frequency of PFS, cetuximab (OR: 1.39; 95% CI: 1.01, 1.91; p=0.042) and nivolumab (OR: 1.81; 95% CI: 1.34, 2.44; p<0.01) were significantly superior to the control. For overall survival (OS), nivolumab (71.6%) in Cox analysis and nimotuzumab (69.7%) in frequency analysis were found to have relative advantages. Nimotuzumab combined with CRT was significantly better than the control with regard to endoscopic and the pathologic complete response (epCR; OR: 2.81; 95% CI: 1.28, 6.14; p=0.011) and objective response rate (ORR; 4.71; 95% CI: 1.45, 15.29; p=0.008). The targeted drugs were not associated with significant SEA risk.

**Conclusion:**

In conclusion, compared to CRT alone, cetuximab and nivolumab combined with CRT were found to significantly improve the PFS rate only based on the frequency results. However, there was no benefit in terms of OS. For epCR and ORR, nimotuzumab was better than the blank control. Considering the limitations in this study, more well-designed RCTs are needed in the future to validate the results.

## Introduction

Esophageal cancer (EC) ranks as the eighth most common cancer type worldwide and the sixth leading cause of cancer-related deaths. In developing countries, it is the fourth leading cause of cancer-related deaths (1, 2). There are two main subtypes, squamous cell carcinoma and adenocarcinoma, which have different epidemiological and biological characteristics (3). In previous reports, more than half of EC patients had locally advanced or metastatic disease at the initial diagnosis (4). Regardless of the histological type, patients with locally advanced or metastatic EC have a poor survival prognosis, with a 5-year overall survival (OS) of less than 15% (2). Therefore, there is still an urgent need for medical advancement to improve the prognosis of locally advanced or metastatic EC patients.

For inoperable locally advanced EC patients, concurrent chemoradiotherapy (CRT) is the preferred treatment strategy (4, 5), and it is even regarded as the standard treatment for inoperable esophageal squamous cell carcinoma (6). In previous meta-analyses, neoadjuvant CRT combined with surgery could increase the radical resection rate, reduce the occurrence of complications, and improve survival prognosis compared to chemotherapy, radiotherapy or surgery alone for esophageal squamous cell carcinoma (7). Compared with other multimodal treatments, neoadjuvant CRT also has advantages in OS and disease-free survival outcomes (8–11).

However, attempts to improve prognosis by improving CRT strategies have failed, such as increasing the radiation dose (12), changing chemotherapy regimens (13), and combining CRT with surgery (14). In the past few years, the use of targeted agents alone or combined with multimodal treatments has been considered a future direction of development. There is evidence that cetuximab combined with multimodal treatments significantly improved the response rate and disease control rate in metastatic EC patients but did not improve OS or progression-free survival (PFS) outcomes (15). A recent systematic review suggested that anti-EGFR agents have beneficial effects on prolonging the OS of EC patients and improving the ORR and DCR, but they will bring adverse effects (16). In addition, for the optimal treatment of resectable EC, a network meta-analysis suggested that CRT combined with surgery is the best option (17). Therefore, whether targeted agents can further improve the CRT effect has become an interesting aspect.

There are currently a variety of targeted agents used in EC treatment. Whether agents combined with CRT have benefits over CRT alone and which agents combined with CRT could bring greater survival or local control benefits remain unclear. This study will comprehensively analyze targeted agents combined with CRT in the treatment of EC by network meta-analysis.

## Methods

This meta-analysis was reported according to the preferred reporting items for systematic reviews and meta-analyses (PRISMA) extension statement for network meta-analysis (18).

### Search Strategy

We identified studies on CRT-related treatment for EC published up to 5 August 2021. Public electronic databases, including PubMed, Embase, and the Cochrane Library, were comprehensively searched. The following keywords were used: “esophageal”, “esophagus”, “gastroesophageal”, “oesophageal”, “neoplasms”, “cancer”, “malignant”, “carcinoma”, “tumor”, “random*”, “randomized”, “chemoradiotherapy”, “chemoradion”, “chemoradio*”, “radiochemotherapy”, and “radiochemo*” (**Supplementary Table 1**). Only English language articles were included. To avoid omission, the references of related reviews were also manually checked.

### Inclusion/Exclusion Criteria

The inclusion criteria were as follows: 1, studies that included EC patients; 2, studies that were of randomized controlled trial (RCT) design; 3, studies that compared targeted agents combined with CRT and CRT alone; and 4, studies that reported one of the following results: OS, PFS, objective response rate (ORR), or serious adverse effects (SAEs). The exclusion criteria were as follows: 1, studies that included gastric cancer patients; 2, studies with CRT regimens that were obviously different between the intervention and control groups; and 3, studies that did not report one of the results described above. Reviews and comments were also excluded.

### Data Extraction

Two authors independently extracted the data from the included studies. The extracted contents included first author, publication year, country, registration, study abbreviation, sample size, age, targeted agent, CRT regimen, and length of follow-up. The major outcomes included OS, PFS, ORR, and SAE. Other results that were reported in more than two included studies were also analyzed. When the hazard ratio (HR) of the OS or PFS results was not provided, the results were obtained by assessing the Kaplan-Meier curve. The Cochrane bias risk tool was used to assess the methodological quality of the included RCTs (19).

### Statistical Analysis

Dichotomous data were combined using the odds ratio (OR) and its 95% confidence interval (CI), and HRs and 95% CIs for survival data were calculated as the overall effect for OS and PFS. The frequentist framework random-effects model was used for mixed multiple treatment comparisons. Global inconsistency was evaluated by the fitness of the consistency model and the inconsistency model, and local inconsistency was assessed by the closed loops in the network comparisons. The surface under the cumulative ranking curve (SUCRA) probabilities were used to rank the treatments for each outcome (20). In addition, hierarchical cluster analysis with ward.D2 method was carried out according to SUCRA. Comparison-adjusted funnel plots were also used to assess the potential small-study effects (21). Subgroup analysis was also performed for populations with different characteristics to explore the role of targeted drugs in patients with different characteristics. Stata software (version 14.0), R program (version 4.1.0), and Review Manager (version 5.3) were used in this work.

## Results

### Literature Search

**Figure 1:**
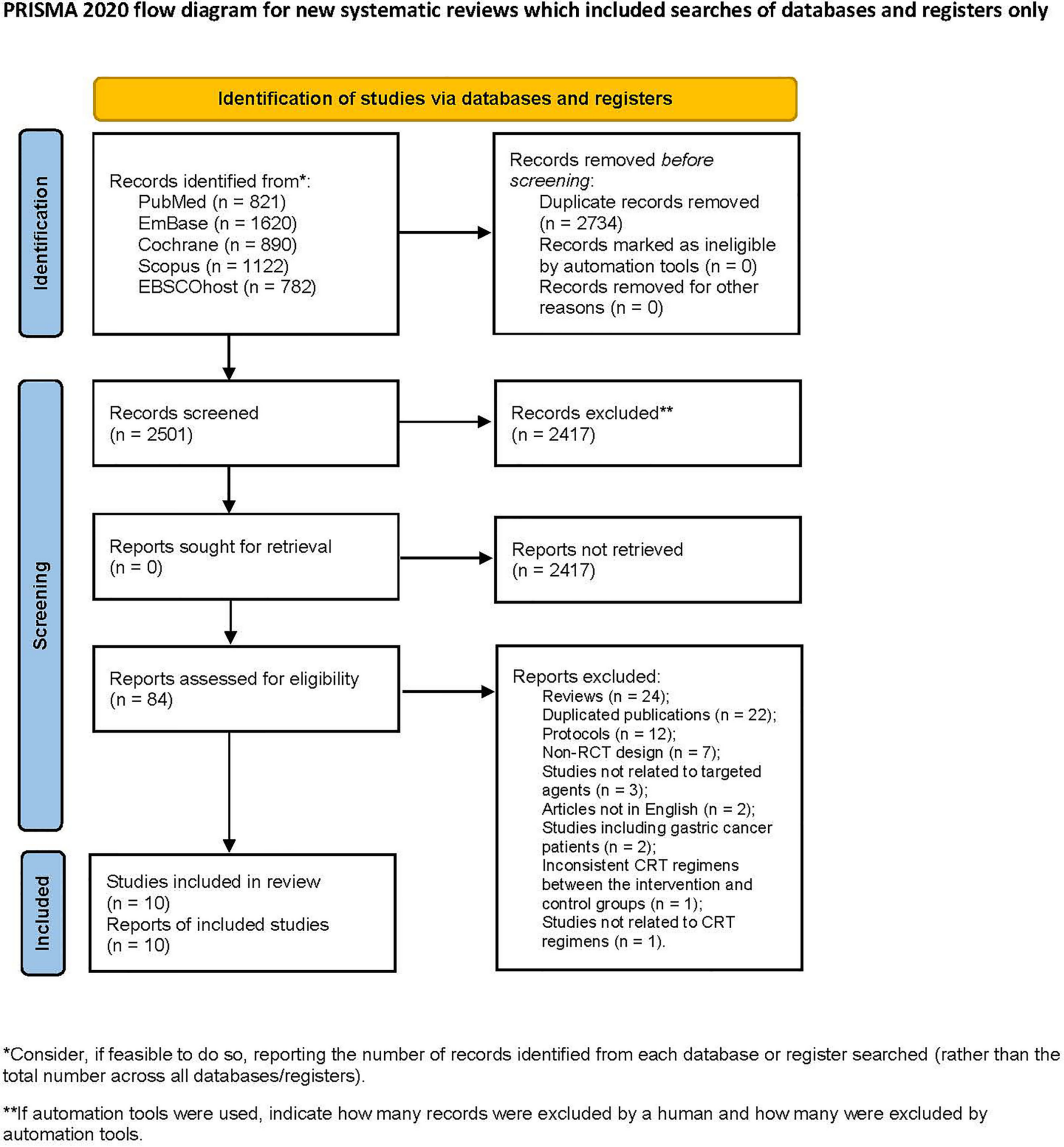
Flowchart illustrating the literature search and the selection of included studies.

A total of 5235 articles were retrieved from the database search, and 2501 articles remained after removing duplications. After screening the titles and abstracts, 2417 articles were excluded. 84 articles were screened in full text. Among them, 74 articles were excluded for the following reasons: reviews (24), duplicated publications (22), protocols (12), non-RCT design (7), studies not related to targeted agents (3), articles not in English (2), studies including gastric cancer patients (2), inconsistent CRT regimens between the intervention and control groups (1), and studies not related to CRT regimens (1). Finally, 10 articles were included in the analysis (22–31), including 2 conference abstracts (29, 30) (**Figure 1**).

### Characteristics of Included Studies

**Figure 2:**
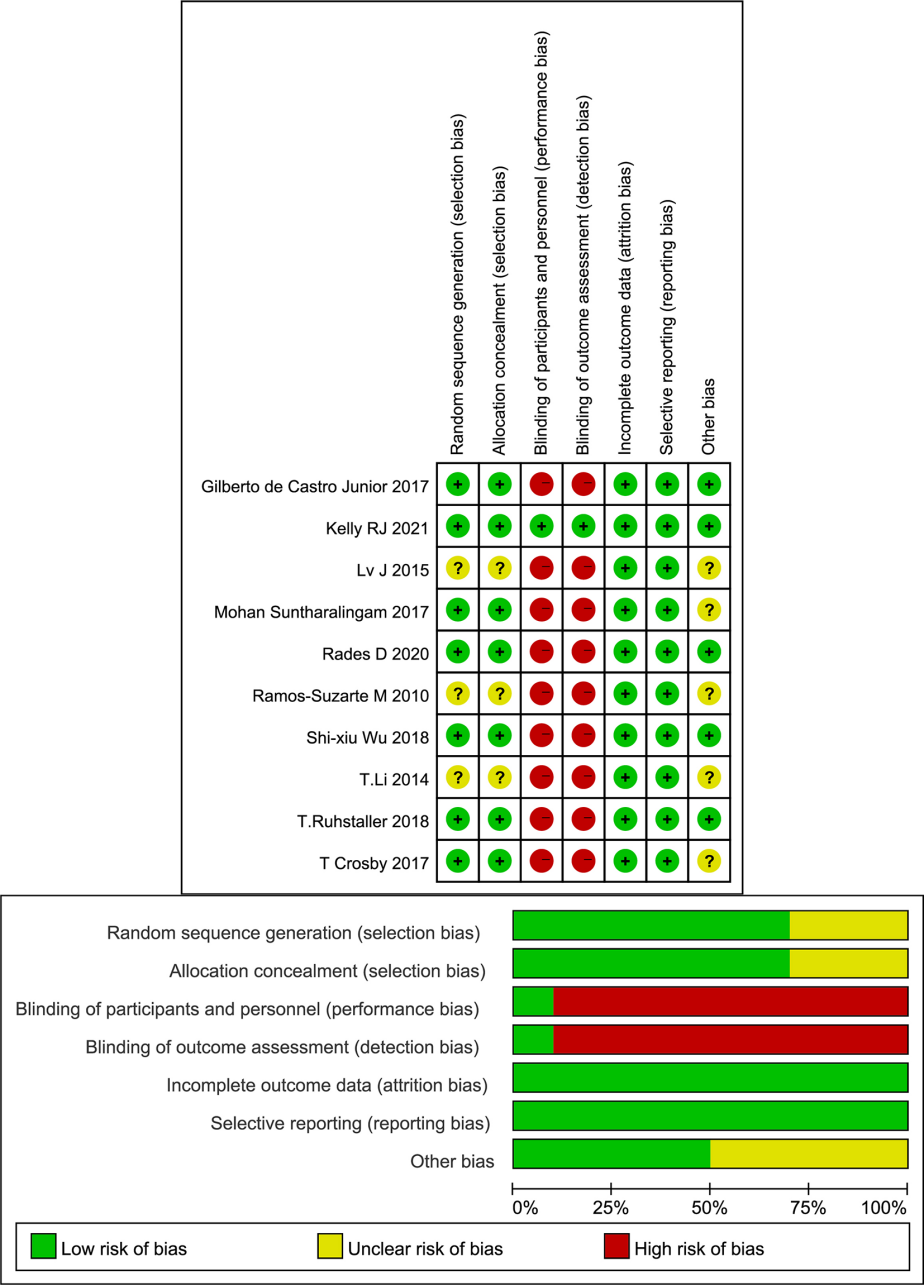
Risk of bias graph for each included study.

The included studies were published between 2010 and 2021, and 8 studies were registered. The two conference abstracts did not specify the age of the patients. Three studies included only squamous cell carcinoma EC patients (24, 29, 30). The analyzed targeted agents included erlotinib, cetuximab, nimotuzumab, nivolumab and endostatin. The main cytotoxic agents used in the CRT regimens included paclitaxel, docetaxel, cisplatin, fluorouracil, and capecitabine (**Table 1**). Although most studies were registered, not all studies adopted a blinded design. Because the main results, such as OS and PFS, were objective, overall, the quality of the included studies was acceptable (**Figure 2**).

**Table 1:** Characteristics of the included studies.

Author	Local	Registration	Abbreviations	Sample size	EC Type	Age, years	Targeted agents	CRT regimens	CRT phase	Total dose of radiation	Follow-up
Kelly et al. (22)	Multilateral	NCT02743494	Checkmate 577	794	**Esophageal or gastroesophageal junction cancer**	62 (26–86)	Nivolumab	Platinum with paclitaxel(or fluorouracil),radiation	Neoadjuvant	45 Gy	45 Months
Rades et al. (23)	Germany	NCT01787006	LEOPARD-II	68	Non-metastatic esophageal carcinoma	64(44-80)	Cetuximab	Cisplatin,fluorouracil,radiation	Definitive	59.4 Gy	2 Years
Xie et al. (24)	China	NCT00686114	NA	352	**Esophageal squamous cell carcinoma**	61(35-69)	Erlotinib	Paclitaxel,cisplatin,radiation	Definitive	60 Gy	10 Years
Ruhstaller et al. (25)	Multilateral	NCT01107639	SAKK 75/08	300	Esophageal carcinoma	61(53-68)	Cetuximab	Docetaxel,cisplatin,radiation	Neoadjuvant	45 Gy	5 Year
de Castro Junior et al. (26)	Brazil	NCT01249352	NICE	107	Locally advanced esophageal carcinoma	59(35.4-81.4)	Nimotuzumab	Cisplatin,fluorouracil,radiation	Definitive	50.4 Gy	Open
Suntharalingam et al. (27)	US	NCT00655876	RTOG0436	344	Esophageal carcinoma	64(32-87)	Cetuximab	Paclitaxel,cisplatin,radiation	Definitive	50.4 Gy	Open
Crosby et al. (28)	UK	ISRCTN:47718479	SCOPE-1	258	Non-metastatic esophageal carcinoma	66.7(IQR:60.9-72.9)#	Cetuximab	Cisplatin,capecitabine,radiation	Definitive	50 Gy	Open
Lv et al. (29)	China	ChiCTR-TRC-13003908	NA	63	**Locally advanced esophageal squamous cell carcinoma**	NA	Endostatin	Docetaxel,cisplatin,radiation	Definitive	60-66 Gy	2 Year
Li et al. (30)	China	NA	NA	37	**Esophageal squamous cell carcinoma**	NA	Endostatin	Docetaxel,cisplatin,radiation	Definitive	60-66 Gy	NA
Ramos-Suzarte et al. (31)	Cuba	NA	NA	68	Advanced esophageal carcinoma	58.8(54.8-62.8)	Nimotuzumab	Cisplatin,fluorouracil,radiation	Definitive	75.6 Gy	NA

NA, not-available; CRT, chemoradiotherapy; EC, esophageal cancer.

^#^interquartile range.

### Results of the Meta-Analysis

**Figure 3:**
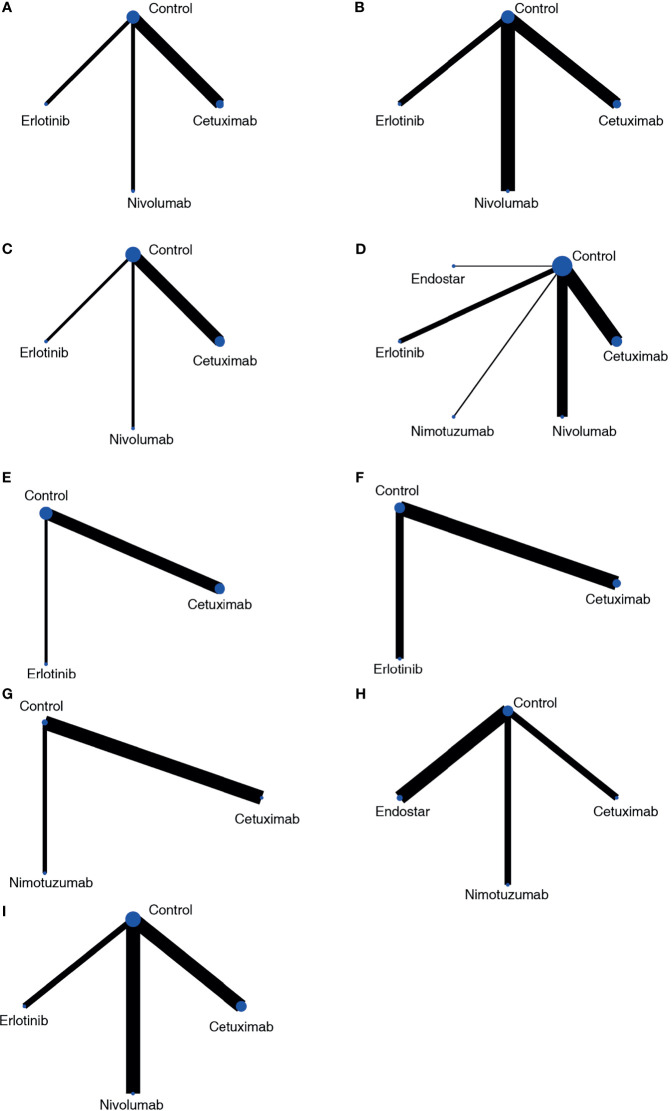
Network plots of the outcomes of targeted agents and blank control combined with CRT in the network meta-analysis. **(A)** PFS based on HR results; **(B)** PFS based on frequency; **(C)** OS based on HR results; **(D)** OS based on frequency; **(E)** locoregional recurrence based on HR results; **(F)** locoregional control rate based on frequency; **(G)** endoscopic and pathologic complete response; **(H)** ORR; **(I)** SAE.

**Figure 4:**
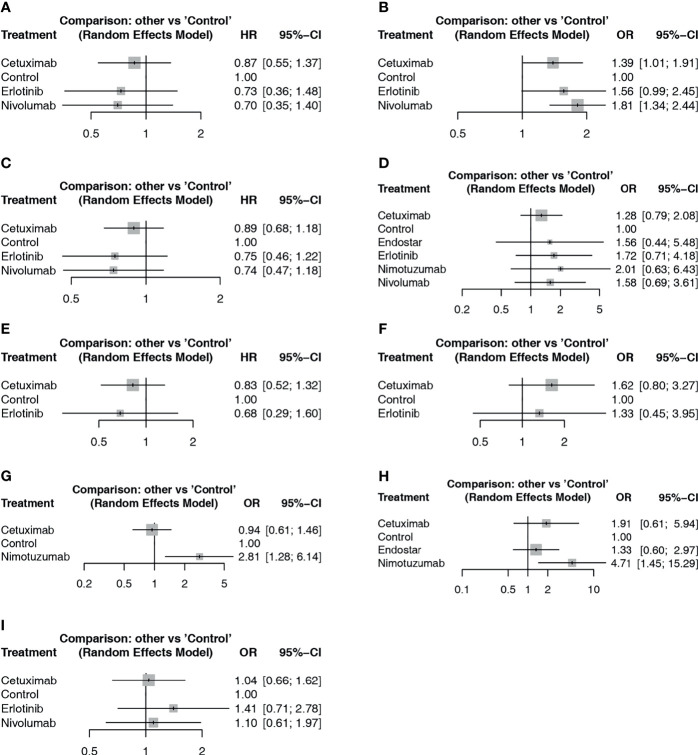
Forest plot of network meta-analysis comparisons between targeted agents and blank control with CRT. **(A)** PFS based on HR results; **(B)** PFS based on frequency; **(C)** OS based on HR results; **(D)** OS based on frequency; **(E)** locoregional recurrence based on HR results; **(F)** locoregional control rate based on frequency; **(G)** endoscopic and pathologic complete response; **(H)** ORR; **(I)** SAE.

For PFS results, studies reported the results from two aspects, including the frequency of PFS until the longest follow-up period and the Cox analysis results reported based on survival analysis. In the network meta-analysis of Cox results, cetuximab, erlotinib, nivolumab and blank control combined with CRT were included (**Figure 3A**). In the pairwise comparisons, no significantly different results were found (**Figure 4A**). The SUCRA ranking showed that nivolumab (67.4%) and erlotinib (64.6%) had relative advantages. Regarding the frequency of PFS (**Figure 3B**), cetuximab (OR: 1.39; 95% CI: 1.01, 1.91; p=0.042) and nivolumab (OR: 1.81; 95% CI: 1.34, 2.44; p<0.01) were significantly superior to the control (**Figure 4B**). SUCRA ranking suggested nivolumab (85.8%) is superior.

For the OS results, in the network meta-analysis of Cox results, cetuximab, erlotinib, nivolumab, and blank control combined with CRT were included (**Figure 3C**). In the pairwise comparisons, no significantly different results were found (**Figure 4C**). The SUCRA ranking results suggested that nivolumab (71.6%) combined with CRT was superior to the other strategies. Regarding the frequency of OS, Endostar, erlotinib, nimotuzumab, cetuximab, nivolumab, and blank control combined with CRT were included in the network meta-analysis (**Figure 3D**). There were no significantly different results in the pairwise comparisons (**Figure 4D**). The SUCRA results showed that nimotuzumab (69.7%) combined with CRT was superior to the other strategies, followed by erlotinib (62.2%) and nivolumab (56.4%).

For the locoregional recurrence (failure) results based on Cox regression analysis, cetuximab, erlotinib, and blank control combined with CRT were analyzed (**Figure 3E**). The pairwise comparisons did not show significant differences (**Figure 4E**). The SUCRA ranking suggested that erlotinib (71.6%) was relatively advantageous. The frequency results of the locoregional control rate at the longest follow-up period (**Figure 3F**) showed that cetuximab combined with CRT was relatively advantageous (76.9%). However, there were no significant differences in the pairwise comparisons (**Figure 4F**).

For the endoscopic and pathologic complete response (epCR) results, cetuximab, nimotuzumab, and blank control combined with CRT were analyzed (**Figure 3G**). In the pairwise comparisons, nimotuzumab combined with CRT was better than the control (OR: 2.81; 95% CI: 1.28, 6.14; p=0.011) (**Figure 4G**). The SUCRA results showed that nimotuzumab (99.3%) had a relative advantage. For the ORR results, endostatin, cetuximab, nimotuzumab, and blank control were analyzed (**Figure 3H**). The pairwise comparisons showed that nimotuzumab combined with CRT was better than the control (OR: 4.71; 95% CI: 1.45, 15.29; p=0.008) (**Figure 4H**). The SUCRA results showed that nimotuzumab (93.6%) combined with CRT had a relative advantage. In the SAE results, erlotinib, cetuximab, nivolumab, and blank control were analyzed (**Figure 3I**). The pairwise comparison results showed no significant differences, and the SUCRA results showed that CRT alone (73.9%) may cause more SAEs (**Figure 4I**). Since there were no loop comparisons, the consistency model was applied in the above analysis, and there were also no small-study effects found in the network meta-analysis.

**Figure 5:**
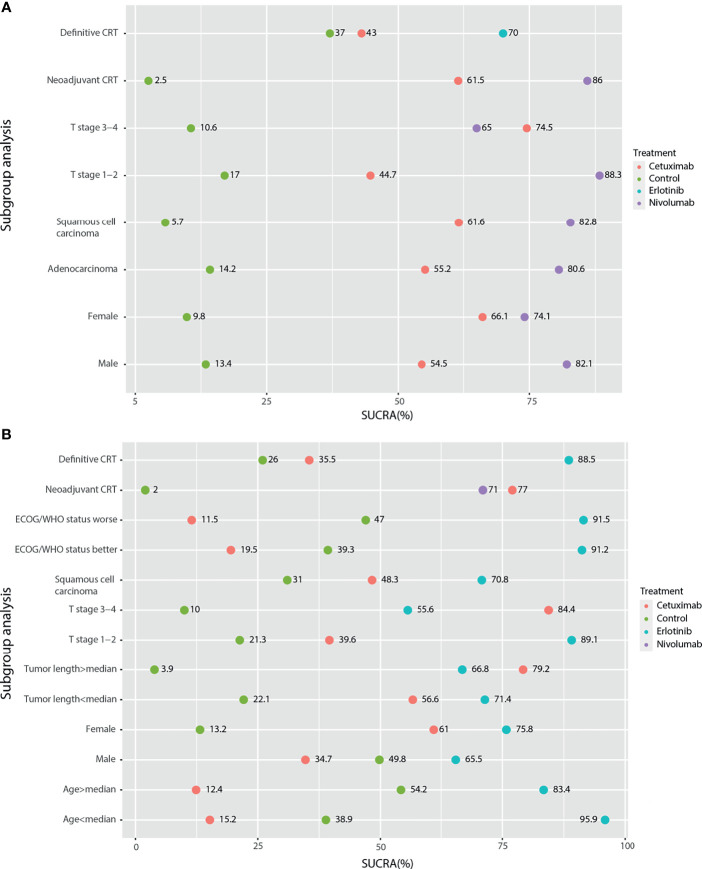
Subgroup analysis of survival outcomes based on the HR results by network meta-analysis. **(A)** PFS; **(B)** OS.

In subgroup analysis, neoadjuvant CRT and definitive CRT were analyzed. For PFS based on Cox results, nivolumab, cetuximab, and the blank control combined with neoadjuvant CRT were assessed, and nivolumab was significantly better than the control (HR: 0.70; 95%CI: 0.57, 0.85; p<0.01), with high SUCRA ranking (86%) (**Figure 5A**). Cetuximab, erlotinib, and the blank control combined with definitive CRT were also analyzed no significantly different results, and SUCRA ranking suggested erlotinib (70%) to be superior. In frequency analysis of PFS, nivolumab combined with neoadjuvant CRT was significantly better than the control (OR: 1.81; 95%CI: 1.34,2.44; p<0.01), with high SUCRA ranking (91%). Erlotinib combined with definitive CRT approached statistical significance (OR: 1.56; 95%CI: 0.99, 2.45; p=0.053) compared to the control.

For OS subgroup results, nivolumab combined with neoadjuvant CRT was significantly better than the control based on Cox analysis (HR: 0.74; 95%CI: 0.60, 0.92; p<0.01), with SUCRA ranking of 71% (**Figure 5B**). No significant differences were found for definitive CRT treatments. Regarding frequency results, nivolumab (OR: 1.58; 95%CI: 1.16, 2.15) and cetuximab (OR: 1.66; 95%CI: 1.05, 2.63; p<0.01) combined with neoadjuvant CRT were both better than the control. For the locoregional recurrence, cetuximab combined with neoadjuvant CRT was significantly better than the control based on both Cox (COX: HR: 0.53; 95%CI: 0.31, 0.90; p<0.01) and frequency (OR: 2.44; 95%CI: 1.46; 4.08; p<0.01) analyses. No significant differences were found for other locoregional recurrence and SAE.

**Figure 6:**
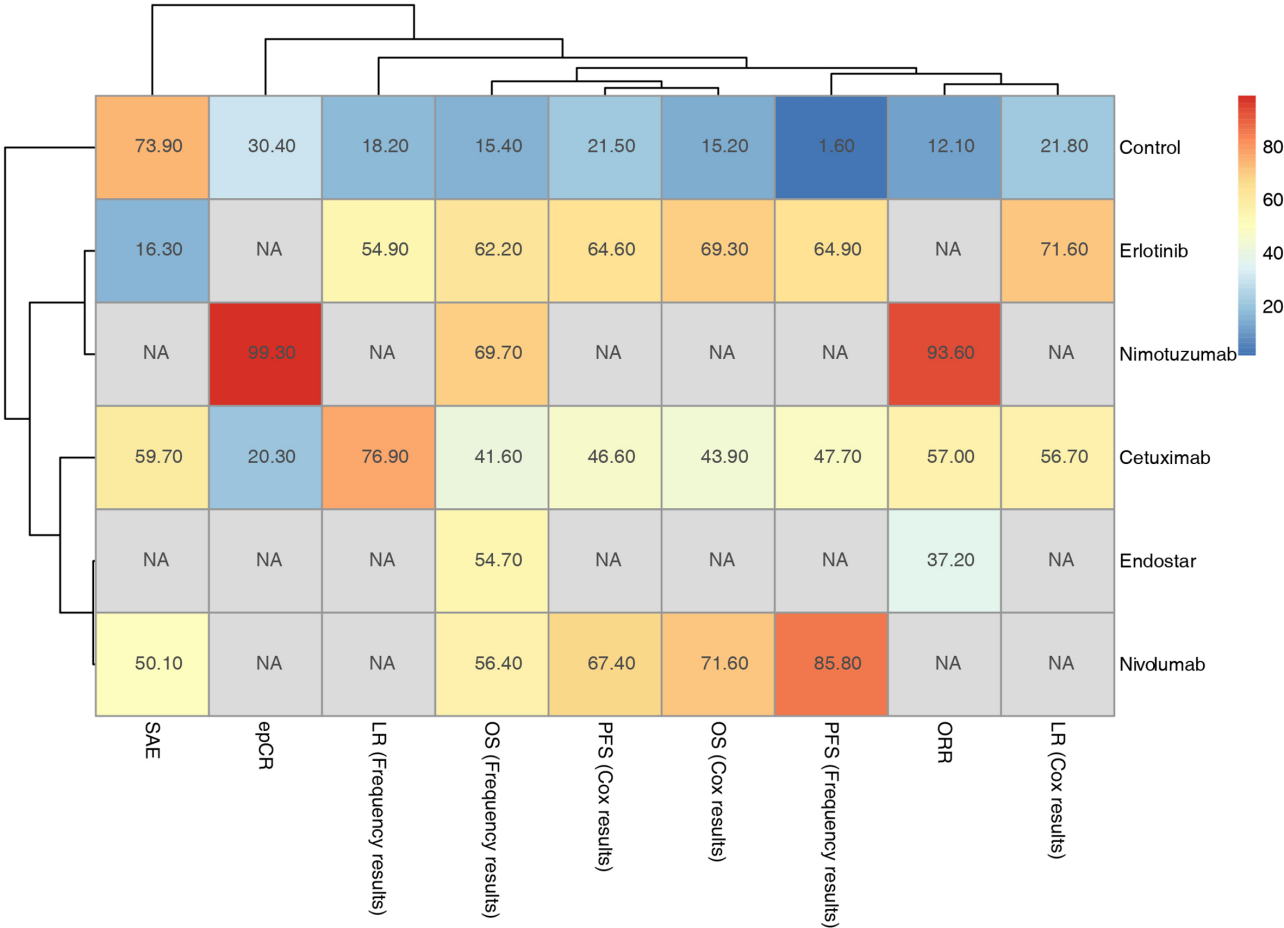
Hierarchical cluster analysis of outcomes for the included targeted agents combined with CRT strategies.

Furthermore, subgroup analysis of PFS based on the Cox regression results showed that in addition to cetuximab, cetuximab had a relative advantage in the T stage 3-4 subgroup; nivolumab had an advantage in the remaining subgroups (**Figure 5A**). For the OS results based on the Cox regression, because there was only a comparative study of cetuximab and the blank control for adenocarcinoma EC patients, it was not included in the subgroup analysis. According to the traditional meta-analysis, there was no significant difference between cetuximab and the blank control in terms of OS (HR: 0.97; 95% CI: 0.71, 1.33; p=0.96). In the squamous cell carcinoma subgroup, the SUCRA results showed that erlotinib (69%) still had a relative advantage, followed by cetuximab (49.3%). According to the subgroup results, when the tumor length was longer than the median and the T stage was 3-4, cetuximab might have a relative advantage. In the other subgroups, erlotinib had a relative advantage. In the sex subgroup, although erlotinib showed a relative advantage, cetuximab was inferior to the control in males but superior to the control in females (**Figure 5B**). Finally, due to the various types of research outcomes, we conducted a clustering analysis of intervention strategies. The results show that erlotinib and nimotuzumab are closely related, mainly based on the frequency-based OS results. It also shows the advantages of nimotuzumab in epCR and ORR. Nivolumab is closely related to Endostar and is also mainly based on frequency-based OS results. However, in the current clustering analysis, there were many missing values, and more studies are needed to correct the results (**Figure 6**).

## Discussion

This work assessed the effect and safety of targeted agents combined with CRT by network meta-analysis. The targeted agents analyzed included erlotinib, cetuximab, nimotuzumab, nivolumab, and endostatin. For PFS, cetuximab and nivolumab combined with CRT could significantly improve the PFS rate compared to the control based on the frequency results. Erlotinib also had borderline statistical significance. It showed therapeutic potential of anti-EGFR and anti-PD-1. For OS results, there were no significant differences between CRT combined with targeted agents and CRT alone, and only nivolumab and nimotuzumab were considered to have relative advantages. For the locoregional control results, only erlotinib and cetuximab were considered to have relative advantages. For the epCR and ORR results, nimotuzumab had advantages. The results also showed that erlotinib, cetuximab, and nivolumab combined with CRT did not cause more SAEs than CRT alone.

In subgroup analysis of PFS, nivolumab had a relative advantage for most population characteristics and when combined with neoadjuvant CRT, and cetuximab had an advantage in T stage 3-4 patients. Considering the subgroup analysis of OS, cetuximab might be more suitable for larger tumor sizes or T stage 3-4 patients.

Previous studies have concluded that cetuximab is not beneficial in patients with low EGFR expression (32), and whether the high expression of EGFR is associated with larger tumors or T stage 3-4 is the reason why cetuximab is more effective can be further confirmed. In addition, sex was an independent predictor of OS in EC patients (33). The sex subgroup results showed that cetuximab combined with CRT was relatively superior to the control in female EC patients but inferior to the control in male EC patients. However, the mechanism is still unclear.

In clustering results, nivolumab combined with CRT can bring more survival benefits, but it lacks results in local control and recurrence. Nimotuzumab has obvious advantages in ORR and epCR. Although only its relative advantage in OS results has been reported, the PFS outcome is still worth looking forward to.

The dysregulation of the expression of inhibitory immune checkpoint molecules is related to tumor evasion from immune surveillance. PD-1 is an immunosuppressive receptor that is highly expressed on activated T cells, B cells and NK cells. The binding of PD-1 to PD-L1 suppresses the T cell receptor and CD28 signaling pathway, resulting in the inhibition of CD8+ T cell activation. PD-L1 expression was observed in 18.4% to 82.8% of ESCC tumors and was related to a poor survival rate (34). In the ATTRACTION-1/ONO-4538-07 study, nivolumab significantly prolonged the OS period (35). Furthermore, when combined with chemotherapy, nivolumab can significantly improve OS and PFS (36), but hyperprogressive disease in elderly people and immune-related pneumonia still need to be considered (37).

EGFR is widely overexpressed in EC patients and is associated with poor prognosis (38, 39). Erlotinib increases the radiosensitivity of tumor cells, showed potential advantages in the OS results and borderline statistically significant in PFS results in our study. In a score-matched analysis of elderly EC patients, compared to CRT, erlotinib combined with radiotherapy had a similar survival prognosis but better compliance and fewer side effects (40). In addition, erlotinib had a certain early effect on squamous cell carcinoma and EGFR-positive patients, but the effect weakened due to reactivation of the MAPK pathway. Therefore, when applying erlotinib, it is necessary to analyze the changes in related pathway activation and even genome expression changes in each patient and further apply other targeted agents (41).

Nimotuzumab shows obvious advantages in ORR and epCR and shows a relative advantage in OS results, but PFS results are lacking. Mechanistically, repeated radiotherapy on EC could cause acquired radioresistance and tumor recurrence, and nimotuzumab could inhibit key tumor survival-related protein, DNA repair, and EGFR signaling pathways, thus reversing acquired radiation resistance and increasing sensitivity (42–44). A retrospective study showed that nimotuzumab combined with CRT can markedly increase the disease control rate and PFS and has a tendency to prolong OS compared to cetuximab combined with CRT for the treatment of locally advanced squamous cell carcinoma (45). However, it should be noted that mutations in the PI3K/AKT/mTOR signaling pathway may cause resistance to nimotuzumab. Periodic mutation analysis will help to adjust more effective drugs (46).

In our study, cetuximab combined with CRT showed obvious advantages in PFS outcome compared to CRT alone. However, one included study reported that the combination of cetuximab and definitive CRT was inferior to definitive CRT alone (with cisplatin and capecitabine in the chemotherapy regimens) (28) in OS results. In addition, the results also showed its relative advantage in the T stage 3-4 subgroup, which may have a guiding role in modifying the applicable population. A previous meta-analysis suggested that cetuximab combined with CRT could improve the 2-year OS of metastatic EC patients, which is different from our results (15). This difference is mainly due to a Chinese study included in the previous study (47). In this Chinese study, based on the control group, the intervention group not only used cetuximab but also used cinobufagin injection, so this study did not meet the inclusion criteria of our work.

This study still had some limitations. Although the inclusion criteria were designed to maintain the consistency of the CRT strategy between the intervention and control groups, the results of the included studies (25, 27, 28) suggested that different CRT strategies among studies may also significantly affect patient outcomes. However, due to the limited number of related studies, it was difficult to further analyze the influence of specific agents, the dosage of cytotoxic agents, and the radiation intensity as well as the extent of their effects on the results by subgroup analysis or other methods. In the included studies, a blinded design was lacking. Some previous studies did not report random sequence generation and allocation concealment, reducing the robustness of the results (29–31). In addition, the range in sample sizes among the included studies was large, and the combinations of small sample sizes resulted in imprecision that substantially reduced the quality of the evidence. Therefore, well-designed large-sample RCTs are needed to investigate the major outcomes in the future.

In conclusion, compared to CRT alone, cetuximab and nivolumab combined with CRT were found to significantly improve the PFS rate only based on the frequency results. However, there was no benefit in terms of OS. For epCR and ORR, nimotuzumab was better than the blank control. Considering the limitations in this study, more well-designed RCTs are needed in the future to validate the results.

## Data Availability Statement

The original contributions presented in the study are included in the article/**Supplementary Material**. Further inquiries can be directed to the corresponding author.

## Author Contributions

PL: Conceptualization, methodology, and writing - original draft preparation. G-FW: Methodology and writing - original draft preparation. HP and LZ: Data curation and software. X-YL and Q-MZ: Software and visualization. QL: Software and validation. J-HZ: Supervision, writing – reviewing, and editing. PL and G-FW contributed equally to this work. All authors contributed to the article and approved the submitted version.

## Conflict of Interest

The authors declare that the research was conducted in the absence of any commercial or financial relationships that could be construed as a potential conflict of interest.

## Publisher’s Note

All claims expressed in this article are solely those of the authors and do not necessarily represent those of their affiliated organizations, or those of the publisher, the editors and the reviewers. Any product that may be evaluated in this article, or claim that may be made by its manufacturer, is not guaranteed or endorsed by the publisher.
