# Supplementary material for: Effectiveness and Safety of Targeted Agents Combined With Chemoradiotherapy for the Treatment of Esophageal Cancer: A Network Meta-Analysis
Source: Front Oncol. 2021 Nov 29;11:621917. doi: 10.3389/fonc.2021.621917 (PMC8666421; doi:10.3389/fonc.2021.621917)
Supplement: Supplementary file 1 [file Table_1.docx]

Supplementary table 1. Detailed search strategy in PubMed database.

| Search number | Query | Results |
| --- | --- | --- |
| 5 | (((#1) AND (#2)) AND (#3)) AND (#4) | 821 |
| 4 | ((random*) OR (randomized)) OR (randomised) | 1,483,522 |
| 3 | ((((chemoradiotherapy) OR (chemoradion)) OR (chemoradio*)) OR (radiochemotherapy)) OR (radiochemo*) | 36,807 |
| 2 | ((((neoplasms) OR (cancer)) OR (malignant)) OR (carcinoma)) OR (tumor) | 5,072,605 |
| 1 | (((esophageal) OR (esophagus)) OR (gastroesophageal)) OR (oesophageal) | 217,157 |
